# A Comprehensive Exploration of Fidelity Quantification in Computer-Generated Images

**DOI:** 10.3390/s24082463

**Published:** 2024-04-11

**Authors:** Alexandra Duminil, Sio-Song Ieng, Dominique Gruyer

**Affiliations:** Department of Components and Systems (COSYS)/Perceptions, Interactions, Behaviour and Simulations of Road and Street Users Laboratory (PICS-L)/Gustave Eiffel University, F-77454 Marne-la-Vallée, France; sio-song.ieng@univ-eiffel.fr (S.-S.I.); dominique.gruyer@univ-eiffel.fr (D.G.)

**Keywords:** image analysis, fidelity scores, datasets, data processing, automated driving systems

## Abstract

Generating realistic road scenes is crucial for advanced driving systems, particularly for training deep learning methods and validation. Numerous efforts aim to create larger and more realistic synthetic datasets using graphics engines or synthetic-to-real domain adaptation algorithms. In the realm of computer-generated images (CGIs), assessing fidelity is challenging and involves both objective and subjective aspects. Our study adopts a comprehensive conceptual framework to quantify the fidelity of RGB images, unlike existing methods that are predominantly application-specific. This is probably due to the data complexity and huge range of possible situations and conditions encountered. In this paper, a set of distinct metrics assessing the level of fidelity of virtual RGB images is proposed. For quantifying image fidelity, we analyze both local and global perspectives of texture and the high-frequency information in images. Our focus is on the statistical characteristics of realistic and synthetic road datasets, using over 28,000 images from at least 10 datasets. Through a thorough examination, we aim to reveal insights into texture patterns and high-frequency components contributing to the objective perception of data realism in road scenes. This study, exploring image fidelity in both virtual and real conditions, takes the perspective of an embedded camera rather than the human eye. The results of this work, including a pioneering set of objective scores applied to real, virtual, and improved virtual data, offer crucial insights and are an asset for the scientific community in quantifying fidelity levels.

## 1. Introduction

In the context of both vision-based and AI-based systems for automated transport systems, it is necessary to address functional evaluation and validation at the level of the algorithms and applications employed by automated mobility systems, as well as in terms of tools, software, and test models, particularly in simulation environments. Therefore, perception is essential and must meet availability and quality requirements. Creating such safe and reliable perception applications with both AI-based and vision-based perception systems poses significant challenges, requiring consideration of numerous variables. Learning-based algorithms for specific tasks, such as semantic segmentation and object detection, are increasingly robust. High-quality, high-fidelity, and high-variety datasets are critical for achieving even more accurate results and enhancing the performance of these algorithms. However, collecting real-world annotated data that capture all these cases is often very time-consuming and difficult. Datasets created from simulated environments are progressively mirroring real-world scenarios with a greater quality. To address this issue, the use of synthetic data has emerged as a viable solution [1,2,3]. Thus, in a simulated environment, it has become possible to incorporate various scenes, actors, lighting, weather simulations, and sensors with ever-improving techniques based on rendering simulators, game engines, and deep learning methods. Moreover, the use of a simulation platform and framework [4,5] is adaptable and efficient for the generation of annotations, ground truths, and data simultaneously, without the need for human intervention. However, there is still a gap between real and simulated data. Indeed, ensuring that synthetic data closely resemble reality is crucial to mitigate the risk of learning-based methods overfitting to unrealistic details.

Part of PRISSMA’s objectives (project involved in the French Grand Challenge on AI) address these issues. Firstly, PRISSMA aims to develop a methodology for the evaluation and validation of systems of systems and AI-based systems. Secondly, PRISSMA also aims to propose a methodology for the verification and validation of tools and models used to carry out the evaluation and validation of applications. To be effective, PRISSMA also proposes procedures for automating the simulation chain, ranging from data and scenario generation, implementation, and execution to the implementation of analysis procedures. Our research fits into this context, and we aim to propose a methodology and metrics to evaluate the quality and especially the fidelity of the synthetic data used in the evaluation and validation process of AI-based systems. The challenge we address consists of proposing a score that provides a quality label to ensure that generated simulation data are sufficiently realistic (from the perspective of a real sensor onboard a vehicle) to be used in the learning, evaluation, and validation stages of perception AI algorithms. With the increase in automated mobility, this topic has become very important and critical.

In [6], a framework of fidelity is proposed, divided into objective and subjective fidelity. Objective fidelity allows for quantification through physical metrics. In our study, fidelity denotes the similarity between features in virtual and real environments. A high fidelity implies a faithful representation, while a low fidelity suggests a simpler one. These terms are commonly used in prior works [7,8], but no quantification methods are proposed. Other works addressing fidelity are primarily application-based, divided into two groups. Some of them focus on quantifying the simulation-to-reality gap (S2R) in sensor models for object detection [9,10,11,12]. They assess sensor performance by comparing real and simulated data, such as point clouds and bounding boxes, to measure the disparity between simulation and reality, validating sensor models. Another group enhances image realism using translation methods, often employing GAN-based architectures [13,14]. Our research introduces novel elements, directly analyzing simulation data features to derive fidelity scores, making it applicable to diverse tasks.

In this work, a set of metrics assessing the level of fidelity of virtual RGB images is proposed. The produced scores will assess if the datasets are faithful enough with respect to the chosen features for evaluating automated vehicle perception. To achieve this, we propose using three feature-based extraction models and a statistical approach based on [15]. Different feature-based extraction models have already been proposed for different applications, with highly satisfactory results [16,17,18].

In Haralick’s work [15], three key elements are identified for image interpretation: spectral, contextual, and textural features. Spectral features capture tonal variations, contextual features stem from data blocks, and textural features denote tonal distribution in a specific band. These features typically characterize the information present in realistic images. Following this, we decided to analyze the texture and frequency features of both synthetic and real images. Specifically, we decided to use texture information from images by employing a statistical texture analysis method known as the gray level co-occurrence matrix method (GLCM) [15]. The GLCM allows one to assess the image structural properties of spacial relation between pixels through a set of 14 Haralick metrics. In addition to this global texture analysis, we have also incorporated the local binary pattern (LBP) method, which provides a more localized texture analysis. Additionally, we have decided to use wavelet transforms to handle high-frequency information, which can be useful for detecting photorealistic images. These methods serve as inputs for convolutional neural networks (CNNs), with the aim of determining the degree of fidelity of synthetic images with respect to the three discussed texture and frequency features. These features provide insight into different aspects of image fidelity. Afterwards, a statistical method is proposed, involving Haralick metrics. In contrast to CNN approaches, we derive fidelity scores with a closed-form equation from the Haralick metrics. The experiments are conducted on data collected in an urban area, under clear daytime weather conditions.

## 2. Related Works

### 2.1. Quality Metrics

Many works focus on generating realistic images with the aim of aligning human perception, and do not take into account the camera’s point of view. A recent work presented in [19] proposes an exploration of metrics and datasets to assess the fidelity of GAN-generated images. Among them, the classical SSIM, PSNR, FID, IS, KID and LPIPS are mentioned. The IS (inception score) metric assess the quality and diversity by computing the Kullback–Leibler divergence between class probabilities of generated and real images. In contrast, the FID (Frechet inception distance) metric quantifies the distance between feature distributions in a high-dimensional feature space for generated and real images. LPIPS (learned perceptual image patch similarity) gauges the perceptual similarity by comparing feature representations with a defined neural network. The SSIM calculates structural similarity based on luminance, contrast, and structure. The KID (kernel inception distance) uses kernel methods to measure the disparity between empirical distributions of features in real and generated images. Ref. [20] proposes an image quality assessment specific to underwater images based on color space multi-feature fusion, involving histogram features, moment statistics, local binary patterns (LBPs), and morphological features. First, underwater images are converted from RGB color to the CIELab color space, which has a higher correlation to human subjective perception of underwater visual quality. The objective is not to quantify the level of fidelity but to quantify the level of quality of underwater images. However, these metrics do not necessarily allow one to measure the difference in terms of perceived realism and fidelity. Moreover, they align with the human perception of image quality, but not with camera perception, and some metrics are strongly dependent on the inception classifier by Google [21]. The authors of [19] recommend combining these measures to get as close as possible to a measure of fidelity of GAN-generated synthetic images, while our proposed study specifically provides fidelity scores. A recent approach [18] introduces a mean for assessing the quality levels of synthetic underwater images. This is achieved by extracting three feature-based measures—statistical, perceptual, and texture-based—from a transmission map. Additionally, the method proposes color features associated with human perception and fractal-based texture features. This work is related to ours since the mentioned method focuses on determining the faithfulness of the underwater images it produces, whereas our objective is to quantify the level of fidelity in computer-generated images.

### 2.2. Simulation-to-Reality Gap Estimation

Recent studies have investigated the S2R gap across various modalities, including camera-based [9,10], RADAR-based [11], and LiDAR-based [12] approaches for object detection algorithms. The S2R gap approach consists of training models using synthetic datasets on the one hand and real data on the other hand. In [9], a comparison between environment simulation software and real-world test drives was conducted, evaluating the gap between simulation and reality using metrics such as precision, recall, MOTA, and MOTP applied to object lists from both domains. Ref. [10] proposes domain adaptation via conditional alignment and reweighting (CARE) to systematically leverage target labels in order to explicitly reduce the gap between simulated and real domains, but it does not offer scores or metrics. Ref. [11] aims to evaluate the fidelity of typical radar model types and their applicability in virtually testing radar-based multi-object tracking with a multi-level testing method. Ref. [12]’s main objective is to quantify the simulation-to-real domain shift by analyzing point clouds at the target level by comparing real-world and simulated point clouds within the 3D bounding boxes of the targets. However, these works were carried out in the context of a specific application, which is object detection and tracking using digital twins. Ref. [17] proposes a method for estimating the S2R gap by computing the Euclidean distance between various real and synthetic datasets. Their approach is similar to ours in that it employs feature embedding methods to extract pertinent features. This method requires the use of existing real datasets to obtain a gap value. This approach is interesting since it does not involve the use of digital twins, as with the previous mentioned methods, which involve more constraints.

### 2.3. Synthetic Dataset Generation

Existing synthetic datasets offer a substantial amount of data covering a wide range of tasks (including object detection, depth estimation, segmentation, instance and panoptic segmentation, and pose estimation) and data from various sensor technologies. In this subsection, we delve into three different methods to generate these synthetic datasets.

#### 2.3.1. Autonomous Driving Simulators for Realism Operating

The use of simulators has brought about remarkable advancements in the field of advanced driving systems. The implementation of multi-agent control in three-dimensional road environments is very challenging. However, it enables the creation of intricate environments with the ability to manipulate various parameters, including lighting, reflections, and weather conditions, in order to simulate real-world conditions with a high fidelity. Some of the notable simulators include the following.

CARLA [5] (CAR Learning to Act) is an open driving simulator designed to facilitate the development, training, prototyping, and validation of autonomous driving models. It has been developed from the Unreal Engine library with a focus on providing a comprehensive platform for simulating urban driving scenarios.

The Pro-SIVIC [4] platform allows for the simulation of vehicle dynamics in a realistic environment using a wide range of sensors such as cameras, GPS, lidar, and radar. It is also possible to control the lighting as well as the weather conditions. This platform uses a LGPL graphical engine (mg engine).

SCANeR [22] is a comprehensive graphical environment that leverages the Unreal rendering engine. It provides users with the ability to configure simulation settings, prepare scenarios, execute simulations, and analyze the obtained results.

Ansys Autonomous Vehicle Simulation [23] was designed specifically to support development, testing, and validation of safe automated driving technologies.

SWEET [24] (Simulating Weather for Intelligent Transportation Systems) is a Monte Carlo simulator developed by the French road planning institute CEREMA. It serves as a research-oriented and physically based simulator designed for internal use. SWEET complements the fog and rain simulation capabilities of the PAVIN platform [25], providing a comprehensive tool set for studying and analyzing weather conditions.

#### 2.3.2. Game Engines for Dataset Generation

Some synthetic datasets have been created with the Unity game engine like virtual Kitti (vKitti) [26] to recreate real-world autonomous driving videos for the digital twins of sequences from the Kitti dataset [27]. The vKitti dataset has been extended to vKitti 2 [3], which provides supplementary data, including diverse weather conditions, and enhances the dataset’s quality.

The Synthia dataset [1], for the synthetic collection of imagery and annotations of urban scenarios, provides a novel virtual world created with the Unity framework. It consists of collection of diverse urban images including RGB images and semantic segmentation.

The GTA V dataset was generated using the open-world video game Grand Theft Auto 5 [2]. It features car perspectives in the streets of American-style virtual cities.

Kitti-Carla is a Kitti-like dataset generated by the CARLA v0.9.1 simulator [28]. The simulated vehicle is equipped with sensors identical to the real Kitti vehicle’s sensors.

#### 2.3.3. Deep Learning for Data Improvement

Several works aim to address the gap between synthetic and real images by improving the photorealism of generated outputs from rendered images. This can be achieved by employing image-to-image translation methods, such as those proposed in [13,14]. Image translation is the ability to learn a mapping function between an input image and a target image in order to transfer it to another domain. In this context, the input image is synthetic and the target is a real-world image. Intel proposed an image enhancement network [13] by generating photorealistic outputs from rendered images. In particular, they extract rendering buffers from their pipeline in order to provide different information about the scene, such as geometry, materials, and lighting. This approach has led to more faithful results when enhancing images from the GTA V dataset. Some works [14] took inspiration from the Cycle-GAN [29] method to enhance the realism or change the style of images into an intricately designed video game. Some other works are very promising, such as DUNIT [30], MGUIT [31], and InstaFormer [32].

Diffusion models [33] have emerged recently, with very impressive results in various tasks, including image generation and image-to-image translation [34]. Some of these models include PALETTE [35] and UNIT-DDPM [36]. There is no specific method tailored to this application yet but these new image-to-image diffusion models outperform the state-of-the-art GAN on several tasks without task-specific architecture customization [35].

## 3. Method

A feature-based analysis of images from different synthetic datasets was conducted in order to study and quantify their degree of fidelity. To characterize this type of image effectively, it is useful to consider specific features such as texture and frequencies. To address high-frequency information, we opted for discrete wavelet transforms. This efficient technique in signal and image analysis allows for the extraction of valuable information at different scales and frequencies from the input data, enhancing our ability to analyze and evaluate the fidelity of images. Texture information, which is also a key feature in images, is dealt with using two complementary approaches: the gray level co-occurrence matrix (GLCM) and the local binary pattern (LBP). The GLCM is often used to characterize the texture of an image by calculating the co-occurrence of pixels within image areas. The LBP is a texture descriptor commonly employed in image texture analysis. Unlike the GLCM, which primarily examines statistical properties, the LBP focuses on local spatial patterns. Employing both of these methods enables an analysis of various aspects of image texture. Moreover, the use of the GLCM allows us to extract the statistical metrics known as Haralick metrics, constituting the second approach.

The resulted pre-processed data are then fed into a CNN network to enable the classification of images as faithful to reality or not. Using feature learning instead of working directly with raw data provides enhanced control over the results while also generating more interpretable and insightful data representations. Indeed, the main hypothesis is that the fidelity calculation is directly dependant of the features. Hence, different types of features must be considered in order to obtain an accurate measurement of a score.

**Figure 1:**
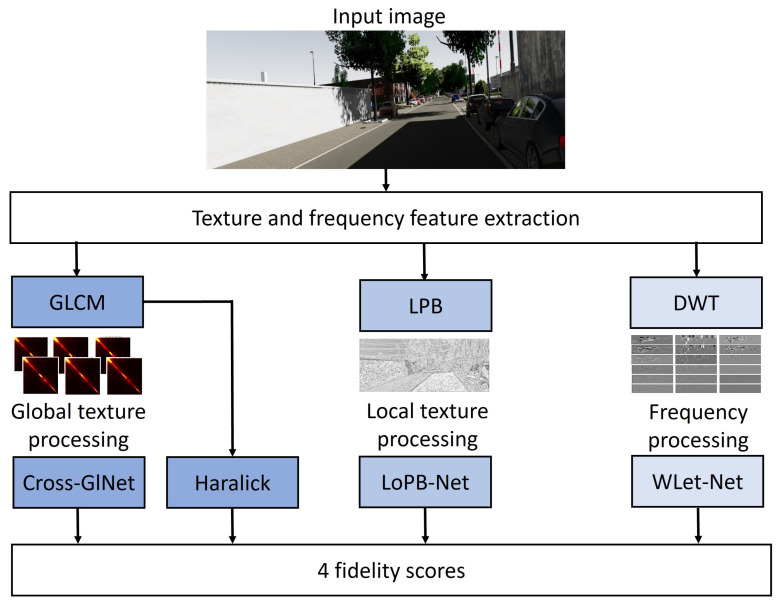
Diagram of the proposed method.

Summarized in Figure 1, our first approach consists of exploring the use of global (GLCM) and local (LBP) representations of the texture and investigates the effectiveness of complementing them with wavelet transforms to emphasize frequency features. The GLCM both serves as an input to the Cross-GlNet model and extracts statistical measures from this matrix known as Haralick metrics.

### 3.1. Dataset Overview

In the context of our exploratory work, datasets are essential. We have collected several well-known and free datasets that are either real or simulated. The real datasets include Kitti [27], Cityscapes [37], ONCE [38], and NuScenes [39], and the synthetic datasets consist of vKitti [3], Kitti-CARLA [28], GTA V [2], and Synthia [1]. In our approach, it is both interesting and desirable to use real and synthetic images which represent the same scenes in order to perform a more efficient and reliable comparison and interpretation. In this context, we selected a clear urban scene consisting of cars, roads, buildings, and vegetation. Finally, we also investigated synthetic data that have been improved from a photorealistic point of view, including GTA V, GTA/Cityscapes and GTA/Mapillary Vistas data. A comparison of these data with the original data is also very interesting for understanding the concept of fidelity in synthetic data and developing algorithms to measure the fidelity scores. All these datasets belong to our custom dataset. For the learning-based methods, each dataset is split into three subsets for algorithm training, validation, and testing; there are 20,572 images in the training set, 6755 in the validation set, and 1000 in the test set.

Each image in the dataset is assigned a label, where the label 0 represents synthetic images and the label 1 represents real ones. The outcomes produced by these networks represent the probability that the images are faithful to reality. Consequently, results approaching 0 indicate a higher likelihood of the images being synthetic, while probabilities closer to 100 suggest that the images are more likely to be realistic. This probability is defined as a fidelity score.

### 3.2. Learning-Based Feature Extraction

In this subsection, we describe the methods employed to obtain texture and frequency features, namely GLCM, LBP, and wavelet transforms.

#### 3.2.1. Gray Level Co-Occurrence Matrices

The GLCM technique provides valuable insights into the co-occurrence of pixels within image regions. Previous studies have successfully used this method to distinguish between GAN-generated images and real images based on their co-occurrence patterns, leading to some very interesting results [40]. In a more specific context, ref. [40] suggests applying six co-occurrence matrices calculated from the R, G, and B channels, as well as the cross-band R + G, G + B, and B + R channels of the images. These matrices are then employed as inputs to a CNN model. Thus, the network can learn to distinguish features between images generated by GANs and those from real images. Inspired by this method, we have chosen to employ GLCMs as a part of the proposed method to quantify the degree of fidelity in images. For this purpose, an analysis of pixel co-occurrence levels was conducted on images from both real and synthetic datasets. This analysis reveals that these two types of images have different characteristics.

**Figure 2:**
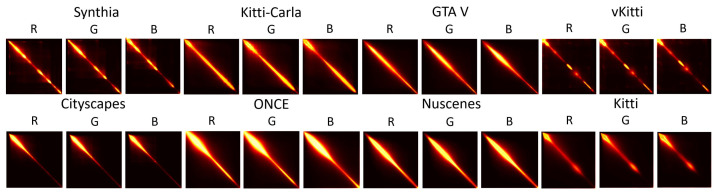
The average of the GLCM was calculated on hundreds of synthetic (**top**) and real (**down**) images from different datasets. Only three of six channels are displayed for more clarity.

Figure 2 illustrates the difference in distribution between synthetic and real images based on the GLCM analysis. As a reminder, the GLCM measures the degree of correlation between pairs of pixels, with the largest values typically being distributed along the diagonal. In the lower part of the synthetic datasets, particularly for the Synthia and the vKitti datasets, the values do not appear to be distributed evenly across the diagonal of the matrices, leading to holes. This could be attributed to a lack of homogeneity and coherence among the objects that constitute the image. With regard to the results of the real images (bottom part of the figure), the values appear to be mainly concentrated in the upper part of the diagonal, indicating a strong presence of co-occurrence without holes. On the other hand, it can be noticed that the GLCMs of the GTA V and Kitti-Carla datasets exhibit a continuous distribution of values along the diagonal. Specifically, some values appear to be concentrated in the upper part of the diagonal, like the GLCM obtained from the real datasets. The lower part of the GLCM presents the aspects found in simulated images. We have chosen to consider the entire matrix and not just the upper part, because it is necessary to enable the network to identify information that may not be perceptible to the human eye.

#### 3.2.2. Local Binary Pattern

The LBP is a texture descriptor that gained popularity in 2003 [41]. It computes the local texture representation of a grayscale image by comparing each pixel with its surrounding neighbors to determine if they have a higher or lower value than the center pixel. This comparison results in a binary output, where a pixel is assigned a value of 1 if it is greater than or equal to the center pixel, and 0 if it is lower. The equation of the $LBP_{P,R}$ is written as:

(1) $$LBP_{P,R}=\sum_{p=0}^{P-1}s\left(g_{p}-g_{c}\right)2^{p},$$

where *R* is the distance between the center and the neighborhood pixels and *P* is the number of neighborhood pixels. $g_{c}$ is the intensity value of the central pixel, $g_{p}$ is the intensity of the neighboring pixel with index *p*, and *s* is a threshold function equal to 1 if x≥0 and equal to 0 if x<0.

**Figure 3:**
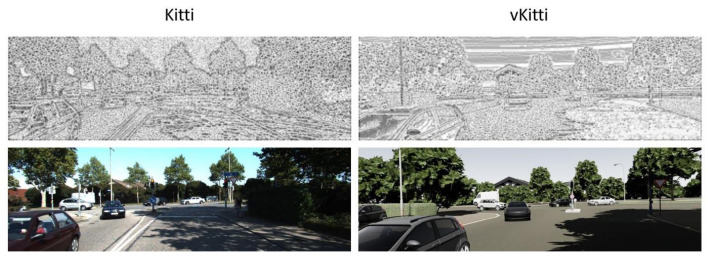
LBP computation on Kitti and vKitti datasets.

Figure 3 presents the visualization of the LBP computation on both the Kitti and vKitti datasets. A circle pattern with a radius of 3 is employed for the computation. A noticeable distinction can be observed between the LBPs of the images captured from the digital twin datasets Kitti (real) and vKitti (synthetic). Specifically, there is a clear disparity in the sky region and the shading of the trees. This method seems to offer a promising approach for distinguishing the characteristics of real images and synthetic images. By incorporating the LBP as a pre-processing step, the CNN can potentially capture and learn distinctive features that differentiate real and synthetic images.

#### 3.2.3. Wavelet Transforms

In our approach, we incorporate a multi-scale discrete wavelet transform (DWT) as one of the inputs of our CNN-based method. Initially, the algorithm estimates the image edges at a broader scale, progressively refining the scale to reveal frequencies within the image. The equation of the DWT [42] is defined as:

(2) $$T\left(a,b\right)=\frac{1}{\sqrt{a}}\int_{-\infty }^{+\infty }x\left(t\right)\psi \left(\frac{t-b}{a}\right)dt,$$

where *a* is the scale parameter, *b* is the location of the wavelet, ψ is a wavelet function, and *x* is the image.

**Figure 4:**
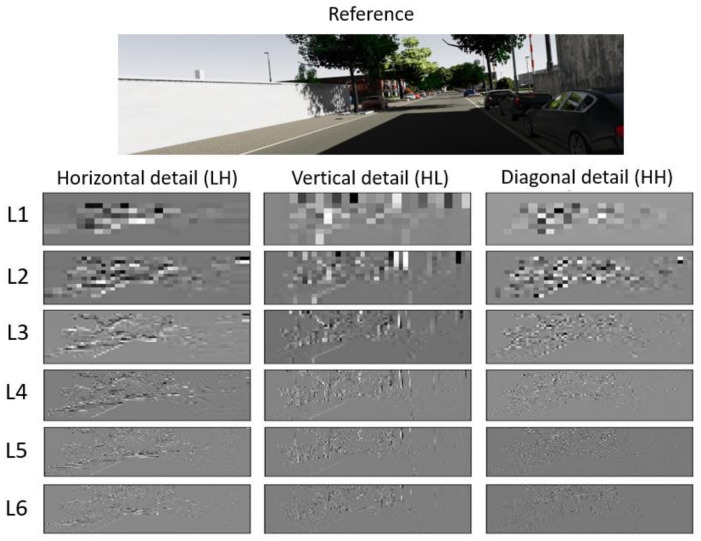
Results obtained after applying the DWT on Kitti images at different levels.

Analyzing the high frequencies in real and synthetic images can provide insights into the textures present in both types of images. Using the two-dimensional DWT may be appropriate in this case. The DWT can transform the resulting image into a combination of four sub-bands, as illustrated in Figure 4, each representing different frequency components: LL (low–low) for image approximation, HL (high–low) for vertical details, LH (low–high) for horizontal details, and HH (high–high) for diagonal details. Figure 4 shows six levels of decomposition using the biorthogonal wavelet function, with orders of (1, 3). Considering all six levels and the HL, LH, and HH components as input from the CNN, this would result in dimensions of 18×W×H. Since finer decompositions are more susceptible to noise, we opted to compute the median absolute deviation (MAD) on each channel. This calculation provides us with an indication of the dispersion of the pixel intensities present in each channel and measures the variability of different levels of the decomposition. Its robustness to outliers makes it reliable for characterizing the data’s variability accurately. Table 1 shows the results. The LL sub-band is not used since it contains an approximation of the original image and lacks substantial information.

Table 1 presents the MAD results of the different frequency components within the six levels. The highest values of MAD indicate a high dispersion of pixels intensities and therefore a high level of noise. Moreover, the first two levels represent low-frequency components involving coarse image details. Ultimately, since our goal is to analyze high-frequency components, we decided to use levels 4, 5, and 6. The sixth level allows us to obtain even finer details than the previous two. Employing a multi-scale approach enables us to incorporate features from early levels later in the process while preventing noise from propagating throughout the network.

### 3.3. Statistical Approach Using Haralick Metrics

The use of Haralick metrics offers an alternative to the models commonly employed for various tasks today. They make it possible to compare the amount of information in simulated images, thereby determining how realistic they are. Indeed, images are composed of both tones and textures, with one often dominating over the other, depending on the image. The predominance of either can significantly impact the results. This is why comparing the metrics on two fully different images may not be useful, as the variations in tonal and textural characteristics can be significantly different. To utilize Haralick metrics with the aim of quantifying fidelity, it may be beneficial to compute the metrics on small patches to obtain textures of isolated regions, like roads or vegetation, to ensure a more important and localized comparison of the Haralick metrics. The Haralick metrics used in the following tables are the angular second moment (ASM), contrast, correlation, sum of squares, variance (Var), inverse difference moment (IDM), sum average (SA), sum entropy (SE), entropy (E), difference variance (DVar), difference entropy (DE), info measure of correlation 1 (IMC1), and info measure of correlation 2 (IMC2). This statistical approach is relevant since it allows us to find a simple formula from the calculations.

## 4. Experiments

### 4.1. CNN-Based Models for Fidelity Score Generation

We propose three sub-networks, namely Cross-GlNet, WLet-Net, and LoPB-Net, that are trained separately in a supervised manner using a custom dataset, as presented in Section 3.1. Each of these sub-networks takes, respectively, GLCM maps, DWT, and LBP maps calculated from RGB images as inputs into CNN networks.

**Figure 5:**
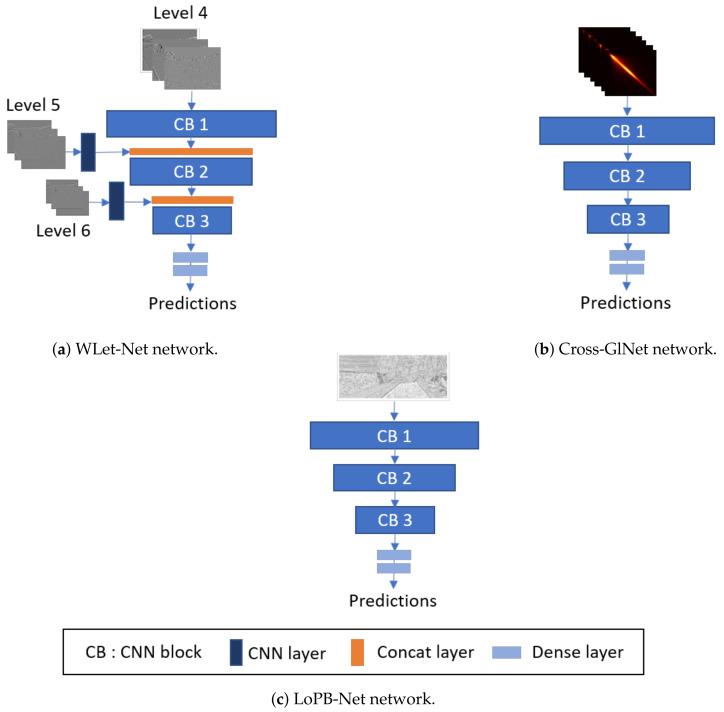
Schematic structure of the three networks.

Inspired by CoNet and Cross-CoNet, Cross-GlNet models use the GLCM or LBP maps as input, but there are some differences in their approach. For clarity, we designate the CNN with GLCM inputs as Cross-GlNet (Figure 5b), and the CNN with LBP inputs as LoPB-Net (Figure 5c). Cross-GlNet computes the GLCM in two directions (horizontal and diagonal) with a pixel distance of 5 on the cross-band RGB channels (R + G, G + B, B + R) of the images. These GLCMs are then stacked together to form an input tensor with size 256×256×6. Only cross-band channels are used in Cross-GlNet, as further experiments demonstrated their superior performance compared to single RGB channels. The models discussed in this section share a nearly identical architecture, as observed in Figure 5. They consist of:

- CB 1: A convolutional layer with 32 filters of size 3 × 3, batch normalization, and ReLu activation followed by a max-pooling layer;
- CB 2: A convolutional layer with 64 filters of size 3 × 3, batch normalization, and ReLu activation followed by a max-pooling layer;
- CB 3: A convolutional layer with 128 filters of size 3 × 3, batch normalization, and ReLu activation followed by a max-pooling layer;
- A dense layer with 256 nodes followed by an ReLu layer;
- A dense layer with one node followed by a sigmoid layer.

The key difference between WLet-Net (Figure 5a) and the two others is the incorporation of multi-scale inputs. As levels 5 and 6 have lower resolutions and finer frequencies than level 4, they are incorporated at a higher stage in the network, fading into a CNN layer with, respectively, 32 and 64 filters of size 1 × 1 followed by a concatenation layer. This type of coarse-to-fine architecture has already been proposed [43] and enables the restoration of high-frequency information through the network. Moreover, incorporating these levels at higher stages seems appropriate since their inputs are noisier compared to those of level 4. For each model, Keras/TensorFlow frameworks are used, employing the SGD optimizer with a learning rate of 0.0001 and using binary cross-entropy as the loss function. The batch size is set to 32, and epochs start at 40, with early stopping to reduce the risk of overfitting.

Table 2 and Table 3 show the fidelity scores (predictions) and accuracy on real and synthetic datasets, respectively. The three models consistently achieve a high accuracy in both scenarios, inspiring confidence in their predictions. Nevertheless, Cross-GlNet exhibits a slight overfitting tendency, which can be attributed partly to the simplicity of the input data. The advantage of employing multiple models lies in their ability to process various aspects of the images, including texture, frequencies, and pixel co-occurrence, ultimately contributing to accurate scores.

The fidelity scores are consistent with the initial hypothesis. Indeed, the real datasets have high scores of fidelity while the synthetic datasets achieved low scores. Those obtained from real datasets provide an indication of the threshold at which images can be considered as realistic. According to Table 2, images scoring 70% or higher can be considered realistic. Nonetheless, training these models can be cumbersome, particularly when it comes to dataset setup and subsequent learning processes. In the following subsection, we employ a more time-efficient approach by calculating Haralick metrics on image patches extracted from various datasets.

### 4.2. Statistic-Based Approach for Fidelity Score Generation

**Figure 6:**
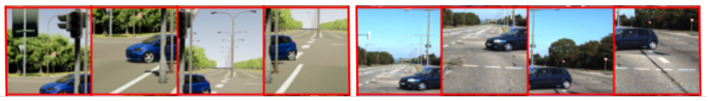
Example of cutting images into patches. **Left**: vKitti patches, **right**: Kitti patches. The patch size used in this figure is 224×224 for more visibility.

In this section, a comparison in terms of Haralick metrics is made between four synthetic and four real datasets. Additionally, another comparison is conducted between GTA V and two enhanced GTA V datasets, which are photorealistic datasets created by Intel [13] from the GTA V dataset. This analysis is applied on 100,000 image patches with a resolution of 64 by 64. The hue channel of the HSV color space was used for this analysis, as this color space allows for better discrimination of image types using Haralick metrics. Min/max normalization is applied to all metrics to ensure that the results are within the range of 0 to 1. Figure 6 illustrates an example of patches cut from the digital twin vKitti and Kitti datasets. The use of small-sized patches enables us to capture various image regions, including roads, vegetation, cars, and buildings.

**Figure 7:**
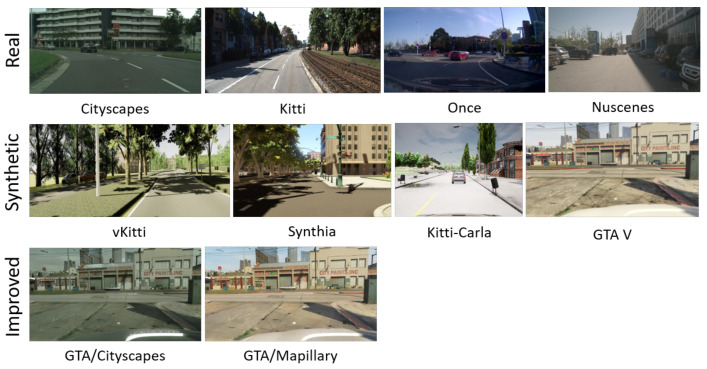
Images from the analyzed datasets.

Table 4 presents Haralick metrics results for four synthetic datasets. These results correspond to the mean values and the standard deviation of metrics, with a total of 100,000 image patches of size 64 by 64 pixels. Figure 7 shows images from the various datasets for a visual comparison. These Haralick metrics have also been computed for the real and improved datasets. At first glance, these results do not provide sufficient information to draw conclusions about which metric is representative or not of the real or synthetic datasets. To deepen the analysis of the results obtained with the Haralick metrics, we propose applying a principal component analysis (PCA). PCA is generally used to reduce the dimensionality of data, but it is also an effective tool for analyzing and interpreting data. This approach is employed to better understand the individual contribution of each metric to the overall information, the links between the various metrics, and their contribution to each principal component (PC). As the first two components contain over 50 % of the data’s information, we focus on the first two PCs for dataset analysis. Then, we compute the contribution of each metric to each principal component $PC_{1}$ and $PC_{2}$ using:

(3) $$K_{i,k}=\frac{c_{i,k}^{2}}{\lambda_{k}},$$

where *k* is the PC index, *i* is the metric index, $\lambda_{k}$ is the eigenvalue associated with the $PC_{k}$, $c_{i,k}$ is the component of the vector $\sqrt{\lambda_{k}}u_{k}$ for the $i^{th}$ metric, and $u_{k}$ is the $k^{th}$ eigen vector.

Table 5 and Table 6 present the computed contribution of each metric to $PC_{1}$ and $PC_{2}$ for different datasets. In Table 5, sum entropy (SE), entropy (E) and difference entropy (DE) metrics contribute equally to $PC_{1}$ for both synthetic and real datasets. These results do not allow us to draw conclusions about the representativeness of the metrics based on the datasets. However, in Table 6, we can observe that some metrics are more significant for the synthetic datasets (highlighted in bold, including correlation, SA, and IMC1), while others (underlined, such as Var and SVar) are more indicative of the real datasets. Table 7 presents the contributions of each metric to $PC_{1}$ and $PC_{2}$ for the improved GTA datasets. The contribution of the metrics to $PC_{2}$, which is characteristic of synthetic images, as shown in Table 6, has noticeably decreased. Specifically, the correlation decreased significantly from 0.15 to 0.038 for GTA/City and to 9e-6 for GTA/Map. Similarly, IMC1 dropped from 0.30 to 0.0005 and 0.056, respectively. As for more informative metrics related to real datasets, Var and SVar increased from 0.11 and 0.12 to 0.26 and 0.26 for GTA/City, and to 0.29 and 0.29 for the GTA/Map dataset. Additionally, there was a notable increase observed in the contrast metric.

**Figure 8:**
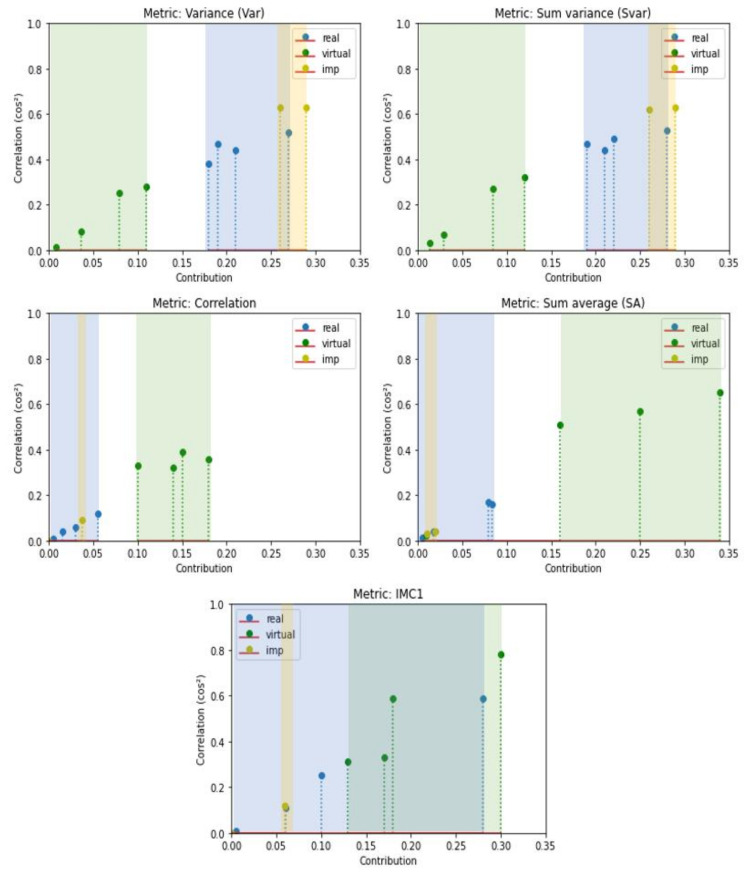
Diagrams of the best metrics’ contributions to PC2.

Figure 8 shows diagrams of the best metrics’ contributions to $PC_{2}$ for all datasets. Notably, the synthetic datasets (green) and the real datasets (blue) are distinctly grouped for each metric, except for the IMC1 metric, where an overlap is observed. A blue dot from the real data is in the middle of synthetic data. The yellow dots, which represent the improved datasets, GTA/Cityscapes and GTA/Mapillary (imp in the legend), are in close proximity to the blue ones, which represent the real data. These observations highlight the possibility of discriminating real and synthetic data and improving the fidelity using specific Haralick metrics.

Based on these figures and the metrics highlighted in bold, some metrics were selected to create a score. The contributions of correlation, SA, and IMC1 metrics to $PC_{2}$, which are representative of synthetic datasets, will be included in the fidelity score as penalties, using the associated correlation contributions. This will place a greater emphasis on the metrics that are representative of real datasets.

## 5. Fidelity Score Results

Several indices need to be considered when quantifying the fidelity of images due to the complexity of real scenes. Indeed, relying solely on Haralick metrics may not be sufficient for establishing an accurate score of fidelity. Therefore, we propose a set of scores including the models described in Section 3.2 and the selected Haralick metrics, which provides a more comprehensive assessment of fidelity. The Haralick sub-score is defined by the following equation:

(4) $$sH=\frac{1}{5}\left(\lambda_{2}K_{Var,2}+\lambda_{2}K_{Svar,2}+\left(1-\lambda_{2}K_{Corr,2}\right)+\left(1-\lambda_{2}K_{SA,2}\right)+\left(1-\lambda_{2}K_{IMC1,2}\right)\right)$$

where $K_{Var,2}$, $K_{Svar,2}$, $K_{Corr,2}$, $K_{SA,2}$, and $K_{IMC1,2}$ are, respectively, the contributions to $PC_{2}$ of Var, Svar, correlation, SA, and IMC1 metrics. $\lambda_{2}$ is the eigenvalue associated with $PC_{2}$. This equation uses the arithmetic average of the correlation contributions $\lambda_{2}K_{i,2}$ of the selected metrics. It takes into account all the best contributions to $PC_{2}$ for both synthetic and real datasets.

Table 8 presents the fidelity scores of synthetic and real datasets employing the different proposed methods, including model-based and Haralick metrics. While the scores obtained from the real datasets (right part of the table) provide valuable information regarding the result consistency, the primary objective is to assess the level of fidelity in the synthetic datasets. The scores obtained for all the datasets reveal a substantial disparity between the synthetic and real data. The evaluated synthetic datasets exhibit a relatively low fidelity. It would then be relevant to evaluate the improved synthetic datasets to analyze the impact of these methods on fidelity scores.

Table 9 presents the scores obtained with the three proposed models and Haralick sH of the enhanced datasets, enabling a comparison with the GTA V scores. A significant improvement is observed across all scores for the enhanced GTA dataset compared to the GTA V dataset. Moreover, adapting this dataset to the style of Cityscapes seems to outperform the Mapillary style. The fidelity scores achieved on enhanced datasets surpass those of GTA V, demonstrating the effectiveness of the photorealism enhancement methods on synthetic datasets [13]. Our proposed method yields promising results, enabling us to quantitatively assess the level of fidelity in synthetic datasets.

**Figure 9:**
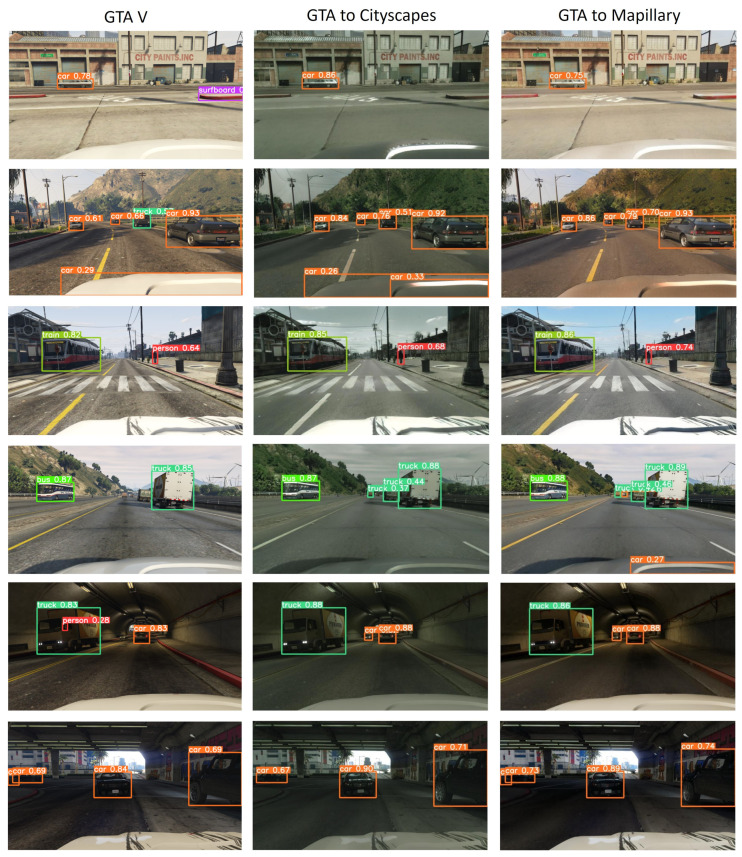
Example of object detection with the YOLOv5 algorithm applied to GTA V and enhanced GTA images.

Fidelity scores give a reliable indication of the detection algorithms’ potential performance. Figure 9 shows images where the YOLOv5 algorithm was used for object detection in both the original GTA V and the enhanced GTA datasets, which should be more faithful versions, as illustrated in Table 9.

Table 10 includes the detection probabilities associated with Figure 9. The detection probability is organized based on the images in each row. The detection probabilities are generally higher for the improved GTA datasets compared to GTA V, particularly in the GTA to Mapillary scenario, where most of them are higher than the rest. However, this is not systematic. For example, in the first row of the table, the probability increases from 0.78 for GTA V to 0.86 for GTA to Cityscapes but decreases to 0.75 for GTA to Mapillary. Furthermore, a few other details should be noted. There are some false positives, such as the detection of the bonnet in the second row of Figure 9, although the detection probabilities remain relatively low (<0.33). In the first row, the false positive in the GTA V image (surfboard) is not detected in the enhanced images.

As the GTA V dataset does not provide the ground truth for object detection, some images are presented as a qualitative evaluation. For quantitative evaluation, we use the vKitti2 dataset and its previous version, which provide ground truths.

Table 11 presents the detection metrics and fidelity scores for two specific scenes (S1 and S18) from the vKitti v1.3.1 and vKitti v2 datasets. The comparison between the two versions is relevant since the second version is a more photorealistic representation of the first. This enhancement is attributed notably to the post-processing refinements implemented in the Unity game engine.

Two types of scenes are used for this comparison. Scene 1 (S1) includes 447 images, while scene 18 (S18) contains 339 images. The precision (P), recall (R), and mAP50 metrics in Table 11 are very useful for assessing the classifier performance, especially in evaluating object detection algorithms. In this context, we aim to demonstrate that higher fidelity scores correlate with the improved performance of detection algorithms. The YOLOv5 algorithm is used in this case. The right part of the table illustrates a correlation between the improved photorealism across different versions of the vKitti dataset and an overall enhancement in fidelity scores. However, it is noteworthy that fidelity scores are slightly higher for version 1 of vKitti in two cases: for the GLCM model in S18 and the LBP model in S1. This kind of result shows the relevance of having several scores based on different sets of features.

To conclude, an overall increase in fidelity scores has a positive impact on the detection performance. This scenario was also applied with both the GTA V and the enhanced datasets (see Figure 9).

## 6. Discussion

**Figure 10:**
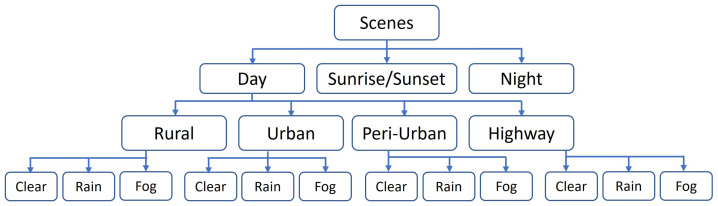
Diagram of various road scenes.

The proposed approach uses several neural networks and metrics to analyze different types of image features in different representative spaces (texture, frequency, co-occurrence). The results showed that the proposed approach makes it possible to distinguish images that are faithful to real images with respect to the chosen extracted features. This identification of significant metrics could be useful in the construction of a model of ‘real’ data fidelity. In the same way, this model could provide advice to modify virtual data and reach a high level of fidelity. However, the data used in this paper were collected only from urban environments under clear weather conditions. The question that arises is how this environment and these weather conditions influence the results provided by our networks and metrics. In future work, it would be valuable to apply these metrics and training processes to various scenes under various weather conditions to further explore their applicability in different contexts. Such an analysis could provide deeper insights into the performance and robustness of the metrics across different scenarios. Road scenes can be divided into four distinct types: rural, urban, peri-urban, and highways, as depicted in Figure 10. These classifications provide a general framework for characterizing different possible environments. Then, each type of scene can be subdivided into different weather conditions: clear, fog, and rain. Taking into account adverse weather conditions is crucial, as they significantly impact scene visibility. In the majority of cases, it is often visually discernible whether an image is computer-generated or not.

Visual cues play a significant role in distinguishing between real and synthetic images. While humans can easily differentiate both types in most cases, expressing the perception of realism mathematically or physically is challenging. The intricacies of human perception make it difficult to define accurate metrics for capturing our intuitive understanding of how faithfully features in synthetic scenes resemble those in real scenes. Nevertheless, visual disparities have been observed, particularly the presence of sharper contours in synthetic images and an overall heightened saturation of colors compared to real images. Eventually, we expect results which will lead us to question the choice of the most suitable features to quantify the fidelity of synthetic images.

## 7. Conclusions

In the development of new automated mobility means, evaluation and validation have become critical stages to assess the performance of components and functions (perception, decision making, path planning, control of actuators) enabling driving automation. Increasingly, these components use AI-based systems and therefore require a time-consuming learning process and a significant number of representative datasets. Since constructing these datasets in real conditions is challenging, it is becoming more common and efficient to use simulation methods to generate the required data. However, when it is necessary to generate synthetic road images covering various road configurations and road situations (urban, suburban, countryside, forest, highway, etc.) with adverse and degraded conditions, there is a lack of effective and, most importantly, objective and rigorous means to assess the level of fidelity of these images in order to use them in training, evaluation, and validation processes. Our work consists of evaluating the characteristics of virtual images as a whole in order to assess their degree of fidelity using a distinct set of metrics which is slightly different from existing methods. Notably, there is no direct comparative method within the existing literature that aligns with our contributions. We propose addressing this critical issue by generating a set of metrics and methods that highlight the variables and parameters quantifying the differences between real and virtual images by comparing some features: GLCM, wavelet transforms, and LBPs. From our study, it appears that quantifying texture information using statistics-based and learning-based metrics such as GLCM, wavelet transforms, LBPs, and Haralick metrics provides valuable insights into such datasets. The use of these two complementary approaches allows us to take advantage of their respective strengths. Model-based methods allow for the analysis of several aspects of an image and take into account the underlying features, despite their computational time demands. In contrast, the statistical approach is much faster, yielding a straightforward equation score despite the limited number of metrics. In addition, the presented method is versatile, making it applicable across diverse applications. Thus, we have proposed the first set of metrics that provide several scores that quantify the level of fidelity of synthetic images. Generating a score is essential to determine whether a virtual dataset will be sufficiently representative and faithful to reality so that is can be used in the learning, evaluation, and validation procedures of AI-based perception systems. This initial work opens the way for the generation of an efficient and objective labeling and certification method for virtual data.

In future work, it would be relevant to deepen our analysis and apply these metrics to different kinds of scenes within each dataset to gather more accurate information. Indeed, the amount of information varies depending on the type of road scene, such as a country road with abundant vegetation or a city road. In addition, investigating different color spaces before applying the feature extraction techniques could be useful for understanding and optimizing our method. To complete our texture-based analysis, it would be valuable to study fractal-based features, which can provide insights into the structural complexity of images.

Our objective was to assign fidelity scores to any synthetic dataset for evaluation purposes. However, evaluating the fidelity in few-shot settings can increase the uncertainty in class assignments, potentially resulting in incorrect classifications of fidelity. To mitigate this uncertainty, various strategies can be employed. In future research, we propose to consolidate the proposed scores with a fusion method in order to take into account the different information sources, as well as the uncertainties and possible conflicts between scores. Additionally, exploring alternative learning-based approaches [44,45] to reduce the possible uncertainties could offer valuable insights.

Understanding the distinguishing characteristics that truly define a realistic image compared to a synthetic image is a topic of significant interest. This information could be used to propose models of what could be “real data” and identify methods of virtual data modification to converge toward more realistic virtual data. It raises the question of what specific attributes really contribute to the perception of realism from a camera point of view.

## Figures and Tables

**Table 1:** Mean MAD of different-frequency components (%) depending on the levels (L).

Components	L1	L2	L3	L4	L5	L6
LH	212	168	133	107	88	75
HL	292	204	152	119	97	82
HH	73	64	52	43	35	30

**Table 2:** Fidelity score predictions/accuracy (%) of Cross-GlNet, WLet-Net and LoPB-Net on real test sets (1000 images).

Real Test Set	Cross-GlNet	WLet-Net	LoPB-Net
Nuscenes	90.94/97.89	98.54/96.06	60.46/61.43
Cityscapes	96.67/99.59	98.05/99.86	87.46/93.00
Once	97.13/99.09	76.05/78.73	63.30/70.13
Kitti	99.81/100	96.85/98.49	94.87/99.20

**Table 3:** Fidelity score predictions/accuracy (%) of Cross-GlNet, WLet-Net and LoPB-Net on synthetic test sets (1000 images).

Virtual Test Set	Cross-GlNet	WLet-Net	LoPB-Net
Synthia	10.42/99.90	3.60/98.94	22.53/78.92
GTA V	12.03/91.90	4.30/97.16	19.08/83.11
Kitti-Carla	0.37/100	1.61/99.14	05.87/98.90
vKitti	0.05/100	51.21/47.97	02.24/99.60

**Table 4:** Haralick metric computation from synthetic datasets, mean (std).

Metric	Synthia	Kitti-CARLA	GTA V	vKitti
ASM	0.224 (0.091)	0.254 (0.047)	0.189 (0.073)	0.239 (0.061)
Contrast	0.102 (0.027)	0.074 (0.026)	0.158 (0.066)	0.146 (0.06)
Correlation	0.575 (0.087)	0.535 (0.071)	0.630 (0.073)	0.695 (0.07)
Variance	0.105 (0.055)	0.093 (0.032)	0.197 (0.084)	0.220 (0.06)
IDM	0.494 (0.054)	0.452 (0.073)	0.467 (0.091)	0.505 (0.076)
S. Average	0.266 (0.102)	0.340 (0.062)	0.416 (0.176)	0.400 (0.09)
S. Variance	0.108 (0.064)	0.094 (0.031)	0.195 (0.085)	0.220 (0.061)
S. Entropy	0.472 (0.056)	0.434 (0.054)	0.508 (0.089)	0.510 (0.069)
Entropy	0.464 (0.061)	0.447 (0.058)	0.498 (0.086)	0.480 (0.069)
D. Variance	0.295 (0.094)	0.280 (0.036)	0.272 (0.105)	0.200 (0.065)
D. Entropy	0.438 (0.072)	0.447 (0.070)	0.451 (0.093)	0.450 (0.066)
IMC1	0.532 (0.065)	0.560 (0.036)	0.579 (0.079)	0.540 (0.049)
IMC2	0.760 (0.072)	0.688 (0.050)	0.756 (0.070)	0.780 (0.061)

**Table 5:** Contribution of each metric to $PC_{1}$. The best contributions are in bold.

Metric	Kitti	City	Once	NuScenes	vKitti	GTAV	Kitti-C	Synthia
ASM	0.11	0.032	0.089	0.10	0.098	0.083	0.094	0.078
Contrast	0.069	0.036	0.063	0.069	0.072	0.070	0.036	0.075
Corr	0.064	0.061	0.012	0.031	0.014	0.0002	0.068	0.027
Var	0.061	0.058	0.065	0.059	0.075	0.073	0.051	0.067
IDM	0.11	0.082	0.12	0.12	0.12	0.12	0.073	0.082
**SA**	0.052	0.011	0.021	$9\times 10^{6}$	0.002	0.004	$5\times 10^{5}$	0.010
SVar	0.048	0.058	0.064	0.055	0.071	0.068	0.052	0.063
**SE**	**0.12**	**0.14**	**0.13**	**0.13**	**0.12**	**0.15**	**0.13**	**0.14**
**E**	**0.12**	**0.13**	**0.12**	**0.13**	**0.12**	**0.14**	**0.12**	**0.13**
DVar	0.032	0.088	0.12	0.075	0.065	0.075	0.074	0.10
**DE**	**0.12**	**0.12**	**0.13**	**0.13**	**0.12**	**0.14**	**0.11**	**0.12**
IMC1	0.0004	0.065	0.028	0.060	0.068	0.0002	0.075	0.025
IMC2	0.08	0.11	0.032	0.039	0.041	0.056	0.10	0.068

**Table 6:** Contribution of each metric to $PC_{2}$. The best contributions among the synthetic datasets are in bold. The best contributions among the real datasets are underlined.

Metric	Kitti	City	Once	NuScenes	vKitti	GTAV	Kitti-C	Synthia
ASM	0.015	0.15	0.095	0.032	0.027	0.040	0.015	0.091
Contrast	0.064	0.10	0.19	0.16	0.009	0.029	0.071	0.020
**Corr**	0.055	0.016	0.030	0.006	**0.18**	**0.15**	**0.14**	**0.10**
Var	0.18	0.19	0.21	0.27	0.008	0.11	0.036	0.079
IDM	0.033	0.12	0.012	0.024	0.006	0.074	0.12	0.13
**SA**	0.018	0.005	0.079	0.084	**0.34**	0.009	**0.25**	**0.16**
SVar	0.22	0.19	0.21	0.28	0.013	0.12	0.028	0.084
SE	0.002	0.011	0.025	0.019	0.0005	0.001	$2\times 10^{5}$	0.009
E	0.014	0.031	0.026	0.026	$4\times 10^{5}$	0.019	0.003	0.036
DVar	0.077	0.088	0.12	0.075	0.056	0.008	0.081	0.005
DE	0.12	0.12	0.13	0.13	0.003	0.020	0.049	0.053
**IMC1**	0.28	0.10	0.005	0.06	**0.17**	**0.30**	**0.13**	**0.18**
IMC2	0.037	0.002	0.084	0.014	0.18	0.11	0.076	0.042

**Table 7:** Contribution of each metric to $PC_{2}$ (improved datasets). The best contributions are in bold and distinct between $PC_{1}$ and $PC_{2}$.

Metric	GTA/City PC1	GTA/Map PC1	GTA/City PC2	GTA/Map PC2
ASM	0.11	0.11	0.043	0.037
Contrast	0.036	0.055	**0.27**	**0.22**
Corr	0.003	0.012	0.038	$9\times 10^{6}$
Var	0.046	0.050	**0.26**	**0.29**
IDM	0.14	0.13	0.013	0.023
SA	0.0008	$2\times 10^{5}$	0.013	0.021
SVar	0.045	0.048	**0.26**	**0.29**
SE	**0.14**	**0.14**	0.023	0.019
E	**0.14**	**0.14**	0.022	0.026
DVar	0.10	0.061	0.002	$5\times 10^{5}$
DE	**0.14**	**0.14**	0.010	0.011
IMC1	0.038	0.071	0.0005	0.056
IMC2	0.039	0.037	0.038	0.009

**Table 8:** Final fidelity scores (%).

Methods	vKitti	GTAV	Kitti-C	Synthia	City	NuScenes	Once	Kitti
GLCM	0.05	12.03	0.37	10.42	96.67	90.94	97.13	99.81
Wavelets	51.21	4.30	1.61	3.60	98.05	98.54	76.05	96.85
LBP	02.24	19.08	05.87	22.53	87.46	60.46	63.30	94.87
sH	34.10	48.14	38.98	41.80	72.76	73.60	72.74	62.42

**Table 9:** Final scores obtained with the enhanced synthetic datasets (%) (GTAV to Cityscapes (GTAV/City) and GTAV to Mapillary (GTAV/Map)) compared to the original GTAV dataset.

Datasets	GTAV	GTAV/Map	GTAV/City
Cross-GlNet	12.03	21.04	42.32
WLet-Net	4.30	29.92	59.63
LoPB-Net	19.08	31.85	43.11
sH	48.14	81.89	82.54

**Table 10:** Detection probabilities associated with Figure 9. The best and second best values are respectively in bold and underlined.

Detection	GTA V	GTA to Cityscapes	GTA to Mapillary
car	0.78	**0.86**	0.75
car	0.61	0.84	**0.86**
car	0.66	0.76	**0.79**
truck/car	0.57	0.51	**0.70**
car	**0.93**	0.92	**0.93**
train	0.82	0.85	**0.86**
person	0.64	0.68	**0.74**
bus	0.87	0.87	**0.88**
truck	nan	0.37	**0.42**
truck	nan	0.44	**0.46**
truck	0.85	0.88	**0.89**
truck	0.83	**0.88**	0.86
car	0.83	**0.88**	**0.88**
car	0.69	0.67	**0.73**
car	0.84	**0.90**	0.89
car	0.69	0.71	**0.74**

**Table 11:** Detection metric calculation (left side) and fidelity scores (right side) for both vKitti versions for two scene types (1 and 18). The best values are in bold.

Datasets	P	R	mAP50	mAP50-95	Wavelet	GLCM	LBP	$s_{H}$
vKitti v1 S1	0.699	**0.488**	0.516	0.238	17.46	38.58	**11.09**	51.44
vKitti v2 S1	**0.766**	0.471	**0.552**	**0.264**	**22.47**	**38.70**	5.97	**57.92**
vKitti v1 S18	**0.969**	0.416	0.486	0.244	06.87	**38.99**	19.96	53.99
vKitti v2 S18	**0.969**	**0.436**	**0.494**	**0.286**	**10.18**	38.81	**29.83**	**80.87**

## Data Availability

vKitti2 dataset: https://europe.naverlabs.com/research/computer-vision/proxy-virtual-worlds-vkitti-2/, accessed on 13 April 2023; Kitti dataset: https://www.cvlibs.net/datasets/kitti/raw_data.php, accessed on 14 April 2023; Once dataset: https://once-for-auto-driving.github.io/, accessed on 7 June 2023; Cityscapes dataset: https://www.cityscapes-dataset.com/, accessed on 19 April 2023; Nuscenes datasets: https://www.nuscenes.org/nuimages, accessed on 12 June 2023; Kitti-Carla datasets: https://npm3d.fr/kitti-carla, accessed on 2 June 2023; Synthia datasets: https://synthia-dataset.net/downloads/, accessed on 11 May 2023; GTA V datasets: https://www.v7labs.com/open-datasets/gta5, accessed on 19 April 2023; [13]’s data: https://github.com/isl-org/PhotorealismEnhancement, accessed on 16 June 2023.

## References

[1] Ros G., Sellart L., Materzynska J., Vazquez D., Lopez A.M. The synthia dataset: A large collection of synthetic images for semantic segmentation of urban scenes. Proceedings of the IEEE Conference on Computer Vision and Pattern Recognition.

[2] Richter S.R., Vineet V., Roth S., Koltun V. (2016). Playing for data: Ground truth from computer games. Proceedings of the Computer Vision–ECCV 2016: 14th European Conference.

[3] Cabon Y., Murray N., Humenberger M. (2020). Virtual KITTI 2. arXiv.

[4] Gruyer D., Pechberti S., Glaser S. (2013). Development of full speed range ACC with SiVIC, a virtual platform for ADAS prototyping, test and evaluation. Proceedings of the 2013 IEEE Intelligent Vehicles Symposium (IV).

[5] Dosovitskiy A., Ros G., Codevilla F., Lopez A., Koltun V. CARLA: An open urban driving simulator. Proceedings of the Conference on Robot Learning, PMLR.

[6] Ye X., Backlund P., Ding J., Ning H. (2019). Fidelity in simulation-based serious games. IEEE Trans. Learn. Technol..

[7] Tu H., Li Z., Li H., Zhang K., Sun L. (2015). Driving simulator fidelity and emergency driving behavior. Transp. Res. Rec..

[8] Zhong Z., Tang Y., Zhou Y., Neves V.d.O., Liu Y., Ray B. (2021). A survey on scenario-based testing for automated driving systems in high-fidelity simulation. arXiv.

[9] Reway F., Hoffmann A., Wachtel D., Huber W., Knoll A., Ribeiro E. (2020). Test method for measuring the simulation-to-reality gap of camera-based object detection algorithms for autonomous driving. Proceedings of the 2020 IEEE Intelligent Vehicles Symposium (IV).

[10] Prabhu V., Acuna D., Liao A., Mahmood R., Law M.T., Hoffman J., Fidler S., Lucas J. (2023). Bridging the sim2real gap with care: Supervised detection adaptation with conditional alignment and reweighting. arXiv.

[11] Ngo A., Bauer M.P., Resch M. (2021). A multi-layered approach for measuring the simulation-to-reality gap of radar perception for autonomous driving. Proceedings of the 2021 IEEE International Intelligent Transportation Systems Conference (ITSC).

[12] Huch S., Scalerandi L., Rivera E., Lienkamp M. (2023). Quantifying the LiDAR Sim-to-Real Domain Shift: A Detailed Investigation Using Object Detectors and Analyzing Point Clouds at Target-Level. IEEE Trans. Intell. Veh..

[13] Richter S.R., AlHaija H.A., Koltun V. (2021). Enhancing Photorealism Enhancement. arXiv.

[14] Mittermueller M., Ye Z., Hlavacs H. (2022). EST-GAN: Enhancing Style Transfer GANs with Intermediate Game Render Passes. Proceedings of the 2022 IEEE Conference on Games (CoG).

[15] Haralick R.M., Shanmugam K., Dinstein I.H. (1973). Textural features for image classification. IEEE Trans. Syst. Man Cybern..

[16] Zhang R., Xu L., Yu Z., Shi Y., Mu C., Xu M. (2021). Deep-IRTarget: An automatic target detector in infrared imagery using dual-domain feature extraction and allocation. IEEE Trans. Multimed..

[17] Gadipudi N., Elamvazuthi I., Sanmugam M., Izhar L.I., Prasetyo T., Jegadeeshwaran R., Ali S.S.A. (2022). Synthetic to real gap estimation of autonomous driving datasets using feature embedding. Proceedings of the 2022 IEEE 5th International Symposium in Robotics and Manufacturing Automation (ROMA).

[18] Li X., Xu H., Jiang G., Yu M., Luo T., Zhang X., Ying H. (2023). Underwater Image Quality Assessment from Synthetic to Real-world: Dataset and Objective Method. ACM Trans. Multimed. Comput. Commun. Appl..

[19] Valdebenito Maturana C.N., Sandoval Orozco A.L., García Villalba L.J. (2023). Exploration of Metrics and Datasets to Assess the Fidelity of Images Generated by Generative Adversarial Networks. Appl. Sci..

[20] Chen T., Yang X., Li N., Wang T., Ji G. (2023). Underwater image quality assessment method based on color space multi-feature fusion. Sci. Rep..

[21] Szegedy C., Vanhoucke V., Ioffe S., Shlens J., Wojna Z. Rethinking the Inception Architecture for Computer Vision. Proceedings of the IEEE Conference on Computer Vision and Pattern Recognition (CVPR).

[22] AVSimulation (2024). SCANeR.

[23] Ansys (2024). Ansys Software.

[24] Ben-Daoued A., Duthon P., Bernardin F. (2023). SWEET: A Realistic Multiwavelength 3D Simulator for Automotive Perceptive Sensors in Foggy Conditions. J. Imaging.

[25] Diao X., Kara M., Li J., Hou K.M., Zhou H., Jacquot A., Amamra A. (2009). Experiments on PAVIN platform for cooperative inter-vehicle communication protocol (CIVIC). Proceedings of the AFRICON 2009.

[26] Gaidon A., Wang Q., Cabon Y., Vig E. Virtual worlds as proxy for multi-object tracking analysis. Proceedings of the IEEE Conference on Computer Vision and Pattern Recognition.

[27] Geiger A., Lenz P., Stiller C., Urtasun R. (2013). Vision meets Robotics: The KITTI Dataset. Int. J. Robot. Res. (IJRR).

[28] Deschaud J.E. (2021). KITTI-CARLA: A KITTI-like dataset generated by CARLA Simulator. arXiv.

[29] Zhu J.Y., Park T., Isola P., Efros A.A. Unpaired image-to-image translation using cycle-consistent adversarial networks. Proceedings of the IEEE International Conference on Computer Vision.

[30] Bhattacharjee D., Kim S., Vizier G., Salzmann M. Dunit: Detection-based unsupervised image-to-image translation. Proceedings of the IEEE/CVF Conference on Computer Vision and Pattern Recognition.

[31] Jeong S., Kim Y., Lee E., Sohn K. Memory-guided unsupervised image-to-image translation. Proceedings of the IEEE/CVF Conference on Computer Vision and Pattern Recognition.

[32] Kim S., Baek J., Park J., Kim G., Kim S. InstaFormer: Instance-Aware Image-to-Image Translation with Transformer. Proceedings of the IEEE/CVF Conference on Computer Vision and Pattern Recognition.

[33] Ho J., Saharia C., Chan W., Fleet D.J., Norouzi M., Salimans T. (2022). Cascaded diffusion models for high fidelity image generation. J. Mach. Learn. Res..

[34] Cheng B., Liu Z., Peng Y., Lin Y. General image-to-image translation with one-shot image guidance. Proceedings of the IEEE/CVF International Conference on Computer Vision.

[35] Saharia C., Chan W., Chang H., Lee C., Ho J., Salimans T., Fleet D., Norouzi M. Palette: Image-to-image diffusion models. Proceedings of the ACM SIGGRAPH 2022 Conference Proceedings.

[36] Sasaki H., Willcocks C.G., Breckon T.P. (2021). Unit-ddpm: Unpaired image translation with denoising diffusion probabilistic models. arXiv.

[37] Cordts M., Omran M., Ramos S., Rehfeld T., Enzweiler M., Benenson R., Franke U., Roth S., Schiele B. The Cityscapes Dataset for Semantic Urban Scene Understanding. Proceedings of the IEEE Conference on Computer Vision and Pattern Recognition (CVPR).

[38] Mao J., Niu M., Jiang C., Liang X., Li Y., Ye C., Zhang W., Li Z., Yu J., Xu C. (2021). One Million Scenes for Autonomous Driving: ONCE Dataset. arXiv.

[39] Caesar H., Bankiti V., Lang A.H., Vora S., Liong V.E., Xu Q., Krishnan A., Pan Y., Baldan G., Beijbom O. nuScenes: A multimodal dataset for autonomous driving. Proceedings of the IEEE/CVF Conference on Computer Vision and Pattern Recognition CVPR.

[40] Barni M., Kallas K., Nowroozi E., Tondi B. (2020). CNN detection of GAN-generated face images based on cross-band co-occurrences analysis. Proceedings of the 2020 IEEE international workshop on information forensics and security (WIFS).

[41] Ojala T., Pietikainen M., Maenpaa T. (2002). Multiresolution gray-scale and rotation invariant texture classification with local binary patterns. IEEE Trans. Pattern Anal. Mach. Intell..

[42] Gilles J. (2013). Empirical wavelet transform. IEEE Trans. Signal Process..

[43] Lelekas I., Tomen N., Pintea S.L., van Gemert J.C. Top-Down Networks: A coarse-to-fine reimagination of CNNs. Proceedings of the IEEE/CVF Conference on Computer Vision and Pattern Recognition Workshops.

[44] Zhang R., Yang S., Zhang Q., Xu L., He Y., Zhang F. (2022). Graph-based few-shot learning with transformed feature propagation and optimal class allocation. Neurocomputing.

[45] Pan X., Li G., Zheng Y. (2024). Ensemble Transductive Propagation Network for Semi-Supervised Few-Shot Learning. Entropy.

